# Chemical Composition, Preliminary Toxicity, and Antioxidant Potential of *Piper marginatum* Sensu Lato Essential Oils and Molecular Modeling Study

**DOI:** 10.3390/molecules28155814

**Published:** 2023-08-01

**Authors:** Bruna de Souza Feitosa, Oberdan Oliveira Ferreira, Suraj N. Mali, Amit Anand, Jorddy Nevez Cruz, Celeste de Jesus Pereira Franco, Sonu Kumar Mahawer, Ravendra Kumar, Marcia Moraes Cascaes, Mozaniel Santana de Oliveira, Eloisa Helena de Aguiar Andrade

**Affiliations:** 1 School of Chemistry, Universidade Federal do Pará, Rua Augusto Corrêa S/N, Guamá, Belém 66075-900, PA, Brazileloisa@museu-goeldi.br (E.H.d.A.A.); 2 Graduate Program in Biodiversity and Biotechnology—Rede Bionorte, Universidade Federal do Pará, Rua Augusto Corrêa S/N, Guamá, Belém 66075-900, PA, Brazil; oberdan@museu-goeldi.br; 3 Department of Pharmaceutical Sciences and Technology, Birla Institute of Technology, Ranchi 835215, India; 4 Department of Chemistry, College of Basic Sciences and Humanities, Govind Ballabh Pant University of Agriculture and Technology, Pantnagar 263145, India; 5 Graduate Program in Chemistry, Universidade Federal do Pará, Rua Augusto Corrêa S/N, Guamá, Belém 66075-900, PA, Brazil; cascaesmm@gmail.com; 6 Programa de Pós-Graduação em Ciências Biológicas—Botânica Tropical, Universidade Federal Rural da Amazônia, Museu Paraense Emílio Goeldi, Av. Perimetral, 1901, Terra Firme, Belém 66077-830, PA, Brazil; 7 Adolpho Ducke Laboratory—Coordination of Botany, Museu Paraense Emílio Goeldi, Av. Perimetral, 1901, Terra Firme, Belém 66077-830, PA, Brazil

**Keywords:** natural products, *Piperaceae*, Amazon, *Artemia salina*, volatile oil, in silico study

## Abstract

The essential oils (OEs) of the leaves, stems, and spikes of *P. marginatum* were obtained by hydrodistillation, steam distillation, and simultaneous extraction. The chemical constituents were identified and quantified by GC/MS and GC-FID. The preliminary biological activity was determined by assessing the toxicity of the samples to *Artemia salina* Leach larvae and calculating the mortality rate and lethal concentration (LC_50_). The antioxidant activity of the EOs was determined by the DPPH radical scavenging method. Molecular modeling was performed using molecular docking and molecular dynamics, with acetylcholinesterase being the molecular target. The OES yields ranged from 1.49% to 1.83%. The EOs and aromatic constituents of *P. marginatum* are characterized by the high contents of (*E*)-isoosmorhizole (19.4–32.9%), 2-methoxy-4,5-methylenedioxypropiophenone (9.0–19.9%), isoosmorhizole (1.6–24.5%), and 2-methoxy-4,5-methylenedioxypropiophenone isomer (1.6–14.3%). The antioxidant potential was significant in the OE of the leaves and stems of *P. marginatum* extracted by SD in November (84.9 ± 4.0 mg TE·mL^−1^) and the OEs of the leaves extracted by HD in March (126.8 ± 12.3 mg TE·mL^−1^). Regarding the preliminary toxicity, the OEs of Pm-SD-L-St-Nov and Pm-HD-L-St-Nov had mortality higher than 80% in concentrations of 25 µg·mL^−1^. This in silico study on essential oils elucidated the potential mechanism of interaction of the main compounds, which may serve as a basis for advances in this line of research.

## 1. Introduction

Aromatic plants are those that possess a pleasant fragrance due to the presence of essential oils in their leaves, flowers, stems, or other parts. These plants are often cultivated for their aromatic properties and are used in various applications, including the culinary, medicinal, perfumery, and food industries. Aromatic plants contain essential oils, which are complex mixtures of volatile compounds. These essential oils (EO) give the plants their characteristic aroma and are extracted through methods such as steam distillation or hydrodistillation. Essential oils possess a wide range of biological properties, including antimicrobial, antifungal, antiviral, and insecticidal activities [1].

Essential oils are composed of a complex mixture of various chemical compounds that give them their characteristic aroma and potential therapeutic properties. While there are hundreds of different compounds found in essential oils, there are some common groups of compounds, such as terpenes, terpenoids, phenylpropanoids, aldehydes, and phenols, among other classes; these compounds are responsible for the potential biological activity of essential oils. In addition, they are responsible for the defense of plants against herbivorous insects and phytopathogens and in the attraction of insects and other pollinators [2,3,4].

Furthermore, the chemical composition of essential oils can be altered by environmental stimuli that can redirect the plant metabolic pathway, causing the biosynthesis of different compounds. These factors prominently include plant/insect, plant/plant, and plant/microorganism interactions; age and development stage; abiotic factors such as temperature, luminosity, rainfall, collection season, and time; and harvest and postharvest techniques. It is worth noting that these factors may correlate with each other and do not act in isolation, though they may exert a joint influence on secondary metabolism [5,6,7]. The chemical characteristics of an essential oil (EO) may vary according to the extraction method, such as hydrodistillation (HD), maceration, solvent extraction, enfleurage, supercritical gas treatment, and microwave-assisted extraction. The heat and pressure used during extraction can, for example, affect the final quality of the EO because the sensitive molecules of a valuable active ingredient can be broken down and oxidized into less effective or sometimes even toxic products [8,9].

The scientific and technological knowledge of *Piperaceae* is considered quite extensive. Chemical studies with *Piper* species have allowed for the identification of a wide variety of new chemical compounds belonging to several chemical classes, including alkaloids, amides, lignans, neolignans, propenylphenols, terpenes, steroids, chalcones, dihydrochalcones, flavones, kavapirones, piperolides, chromenes, and benzoic acid derivatives [10,11,12,13,14,15]. Many of these species are biologically active and have antitumor, antimicrobial, antifungal, antioxidant, insecticidal, and larvicidal potential [16,17,18,19,20,21,22,23]. The species *Piper marginatum* has a diverse chemical composition that depends on the place of collection [24]; however, previous studies do not report the potential toxicity of its essential oils [25,26].

Toxicity tests are designed to evaluate or predict the toxic effects on biological systems and measure the relative toxicity of substances [27]. *Artemia salina* is often used in preliminary toxicity assays due to its sensitivity to various chemical substances [28], including compounds present in essential oils [29]. While the *A. salina* lethality test is commonly used for preliminary toxicity screening, it is important to note that the results obtained from this test may not directly translate to the potential toxicity of essential oils in humans or other animals [30,31,32]; however, they can bring a potential toxicity perspective to natural products. Furthermore, the toxicity in *A. salina* may be related to the inhibition of acetylcholinesterase (AChE) [33], which is an enzyme that plays a crucial role in the termination of nerve impulse transmission by catalyzing the hydrolysis of the acetylcholine neurotransmitter; several studies reported AChE as a potential molecular target to cause the death of *A. salina* using molecular modeling, which can be a useful tool to analyze the potential interactions between the molecules present in essential oils and AChE [34].

Molecular modeling refers to the computational techniques used to study and predict the behavior and properties of molecules. In the context of studying acetylcholinesterase (AChE) in *A. salina*, molecular modeling can be used to gain insights into the structure and function of the enzyme. As the experimentally determined structure of AChE in Artemia salina is not available, molecular modeling techniques can be used to predict its structure. Molecular docking simulations can be performed to study the interactions between AChE and ligands. Molecular dynamics simulations can provide insights into the dynamic behavior of AChE, supporting experimental studies [35,36,37,38].

Additionally, antioxidant analysis methods are important because antioxidants can protect the biological system against the harmful effect of processes or reactions that can cause excessive oxidation [39]. The growing epidemiological evidence regarding the role of antioxidant foods in the prevention of certain diseases has led to the development of a large number of methods to determine antioxidant capacity [40]. Thus, the objective of this study was to study the chemical composition, antioxidant potential, and preliminary toxicity and perform an in silico study to elucidate the potential mechanism of molecular interaction of the major compounds of EOs and volatile concentrates from the leaves, stems, and spikes of *P. marginatum* sensu lato.

## 2. Results and Discussion

### 2.1. Yields of Essential Oils

The essential oil (EO) yields of *P. marginatum* are presented in Table 1. The yields of *P. marginatum* EOs obtained from the leaves and stems by hydrodistillation (HD) and steam distillation (SD) in the months of November and March ranged from 1.66% to 1.83%. The EO yields showed significantly different results; this difference may be related to the seasonality of collection, as described in seasonal studies of the EO of *P. marginatum* [41].

### 2.2. Chemical Composition

In addition to the great qualitative variability in the secondary metabolites among the EOs and volatile concentrates of *P. marginatum* (Table 2), variations in these metabolites were found with respect to the part being studied (leaves, stems, and spikes) and the extraction technique (HD, SD, and simultaneous distillation and extraction (SDE)), as shown in Table 2. Circadian rhythm, humidity, atmospheric air composition, herbivory, and pathogen attack, altitude, ultraviolet and visible radiation, rainfall index, availability of macro- and micronutrients, seasonality, plant age, and temperature were shown to be key factors explaining the quantitative and even qualitative variation in the production of secondary metabolites in the same species [41].

The classes phenylpropanoids (37.93–69.5%), phenylalkanoids (21.1–33.1%), and hydrocarbon sesquiterpenes (4.9–14.7%) were predominant in the EOs of all parts of the plant studied in the present study (Table 2). The main constituents were (*E*)-isoosmorhizole (22.1–32.9%), isoosmorhizole (1.6–24.5%), isomer-2-methoxy-4,5-methylenedioxypropiophenone (1.6–14.3%), and 2-methoxy-4,5-methylenedioxypropiophenone (9–19.9%). Costa et al. [42] evaluated the chemical composition of the EOs of the dry leaves of *P. marginatum* and found the following main constituents: isoelemicin (21.7%), apiol (20.1%), and δ-guaiene (16.7%). Santana et al. [43] identified (*E*)-methyl-isoeugenol (27.08%), (*E*)-anethole (23.98%), and (*Z*)-methyl isoeugenol (12.01%) in the oils of the fresh leaves of *P. marginatum*. The EO from the roots of *P. marginatum* was studied by Hurtado et al. [44] and was characterized by €-anethole (10.10%), (Z)-anethol (8.01%), and safrole (5.78%).

A circadian study of the EO of the leaves of *P. marginatum* showed the strong presence of phenylpropanoids, especially the compound (*Z*)-asarone (33.8–0.2%) and its isomer (*E*)-asarone (20.6–0.2%), in collections performed at different times and under different temperatures and relative humidities [41]. In the study by Souto et al. [45], the EO of the leaves and stems of *P. marginatum* showed two chemical types, A and B; type A was characterized by p-mentha-1(7),8-diene (39.0%), 3,4-methylenedioxypropiophenone (19.0%), and (*E*)-β-ocimene (9.8%), and type B was characterized by (*E*)-isoosmorhizole (32.2%), (*E*)-anethole (26.4%), isoosmorhizole (11.2%), and (Z)-anethole (6.0%). In addition, it is important to mention that phenylpropanoid compounds were highlighted in several studies of the EO of *P. marginatum* [43,46,47].

Da Silva et al. [48] found 3,4-methylenedioxypropiophenone (21.8%), elemol (5.9%), β-caryophyllene (5.0%), and 2-methoxy-4,5-methylenedioxypropiophenone (4.8%) in the EOs of the leaves and dry stems of *P. marginatum* collected in the Amazon. Andrade et al. [24] classified specimens of *P. marginatum* collected in the Amazon into seven chemotypes. 2-Methoxy-4,5-(methylenedioxy) propiophenone, methoxy-4,5-(methylenedioxy) propio-phenone isomer, and trans-isoosmorhizole came from samples collected in the cities of Belém, Pará state (PA), Brazil, while chemotype (*E*)-isoosmorhizole bears a resemblance to the present study; we can observe that this species has a large chemical variability, as shown in the Supplementary Materials, Figures S1–S6, where the ion chromatograms of the different fractions of essential oils and aromas can be found.

**Table 2:** Chemical composition of the aromatic compounds and essential oil of *P. marginatum* lato sensu; **SDE**: simultaneous distillation–extraction; **HD**: hydrodistillation; **SD**: steam distillation; **L**: leaves; **St**: stems; **s**: spikes; **Nov**: November; **Mar**: March (concentration in area relative to percentage).

*Piper marginatum*								
			(SDE)			(HD)		(SD)
Constituents	* IR_L_	** IR_C_	L-St-Nov	L-St-Mar	s-Mar	L-St-Nov	L-St-Mar	L-St-Nov
(2*E*)-Hexenal	846	846		0.2				
α-Pinene	932	932		1.9	3.3	2.1	0.4	
Camphene	946	946		0.2			0.1	
Sabinene	969	969		1.2	2.2		0.5	
β-Pinene	974	974	1.8	0.4	0.5	1.8	0.2	
Myrcene	988	988	1.6					
δ-3-Carene	1008	1001		0.3			0.2	
δ-2-Carene	1001	1008				0.9		
Limonene	1024	1024		0.1	0.2	0.2	0.1	
(*Z*)-β-Ocimene	1032	1032		1.3	1.1	0.7	0.8	
(*E*)-β-Ocimene	1044	1044		2.4	2.4	1.4	1.5	
Terpinolene	1086	1083				0.1		
Linalool	1095	1095		1.9	2.6	0. 2	0.9	0.1
Allo-ocimene	1128	1125		0.8	0.7	0.6	0.4	
(*E*)-Pinocarveol	1135	1135		0.1	0.1		0.04	
(*E*)-Verbenol	1140	1140		0.1	0.1		0.1	
Camphor	1141	1141				0.2		
Isoborneol	1155	1155		0.1		0.1	0.04	
*p*-Mentha-1.5-dien-8-ol	1166	1166		0.1	0.1			
Naphthalene	1178	1178		0.1	0.1			
Methyl chavicol	1195	1195		0.2	0.1	0.1	0.1	0.1
(*Z*)-Anethole	1249	1249	6.8	2.1	0.3	0.6	1.4	0.5
(*E*)-Anethole	1282	1282	1.1	2.9	2.6	3.3	5.4	2.8
Safrole	1285	1285		0.2	0.2			
δ-Elemene	1335	1335	0.7	2.2	0.4	1.5	2.4	1.4
α-Cubebene	1345	1345			0.1			
α-Ylangene	1373	1363		0.3	0.1	0.4	0.3	0.3
α-Copaene	1374	1374	0.7	1.1	4.9	1.1	1.5	0.8
β-Bourbonene	1387	1387		0.1	0.1	0.1	0.1	0.1
β-Cubebene	1387	1387		1.1	0.2		1	
β-Elemene	1389	1389			0.8	0.9		0.9
Methyl eugenol	1403	1403		0.2	0.03		0.2	0.1
β-Caryophyllene	1417	1413	2	3.8	3.2	2.8	3.8	2.4
γ-Elemene	1434	1425		1.2	0.1		0.4	
β-Copaene	1430	1430				0.5		0.5
α-Guayene	1437	1431		0.1			0.1	
Aromadendrene	1439	1440		0.1		0.1		0.1
Isoosmorhizole	1466	1452	24.5	15.2	1.6	13.1	14.2	14.8
Croweacin	1457	1457	3.2	1.1	0.8	2.3	1.2	2.3
trans-Cadina-1 (6), 4-diene	1475	1467						0.2
γ-Gurjunene	1475	1475		0.1		0.2		0.1
γ-Muurolene	1478	1478	0.8	0.2	0.2	0.5		0.4
Germacrene D	1484	1484		0.2	0.3	0.2		0.2
β-Selinene	1489	1489		0.7	0.2		0.7	0.1
(*E*)-Methyl-isoeugenol	1491	1491		0.2	0.2		0.2	
δ-Selinene	1492	1492				0.5		0.6
(*E*)-Muurola-4 (14),5-diene	1493	1493				0.2		0.2
Bicyclogermacrene	1500	1500	0.7	2.4	1	1.3	2.5	1.4
α-Muurolene	1500	1500		0.5	0.04	1	0.6	1
β-Dihydro agarofuran	1504	1503		0.1	1	0.1		0.2
(*E*)-Isoosmorhizole	1517	1504	32.9	19.4	29.8	22.1	23.3	24.1
Cubebol	1514	1508				0.3		0.3
γ-Cadinene	1513	1509						0.8
δ-Cadinene	1522	1513		0.5	0.6	0.8	0.7	
2.4-Dimethoxybenzaldehyde	1526	1522		0.1	0.1			
3,4-(Methylenedioxy)propiophenone	1545	1523		0.1				
Elemicin	1555	1555		0.9		0.7	0.8	0.8
Germacrene B	1559	1559		0.1				
(*E*)-Nerol idol	1561	1561		0.5	0.04	0.2	0.5	0.2
(*E*)-Isoelemicin	1568	1568		0.1	0.2	0.2	0.1	0.2
Spathulenol	1577	1569	0.9	1.9	2.6	1.1	1.4	1.8
Junenol	1618	1618		0.1	0.4		0.1	
(*Z*)-Asarone	1616	1619		0.1	0.3	0.6	0.1	0.6
isomer-2-Methoxy-4.5-methylenedioxypropiophenone	1635	1625	11.6	13.9	1.6	12.9	12.4	14.3
Exalatacin	1655	1640				0.3		0.5
β-Eudesmol	1649	1647		0.8	1	0.6	1	0.9
Selin-11-en-4α-ol	1658	1658		0.2	0.3	0.1	0.2	0.2
Intermedeol	1665	1659				0.2		0.4
(*E*)-Asarone	1675	1675	1	1.4	1.8	1.9	1.3	1.9
2-Methoxy-4,5-(methylenedioxy)-propiophenone	1713	1700	9.5	9	19.9	16.3	12.7	18.8
Monoterpene hydrocarbons			3.4	10.9	13	7.8	5.1	0.1
Oxygenated monoterpenes			0	0.4	0.3	0.3	0.18	0
Hydrocarbon sesquiterpenes			4.9	14.7	12.24	12.1	14.1	11.5
Oxygenated sequiterpenes			0.9	3.6	5.34	2.6	3.2	4
Arylpropanoids			0	0	0	0.3	0	0.5
Phenylalkanoids			21.1	22.9	21.5	29.2	25.1	33.1
Phenylpropanoids			69.5	44.1	37.93	44.9	48.3	48.2
Others			0	0.1	0.1	0	0	0
Total			99.8	96.6	90.51	97.2	95.98	97.4

* RI_L_, retention index in the literature [49,50]; ** RI_C_, retention index calculated from a homologous series of n-alkanes (C_8_-C_40_) in a DB5-MS column. Relative area (%) calculated based on the peak areas.

#### Multivariate Analysis

**Figure 1:**
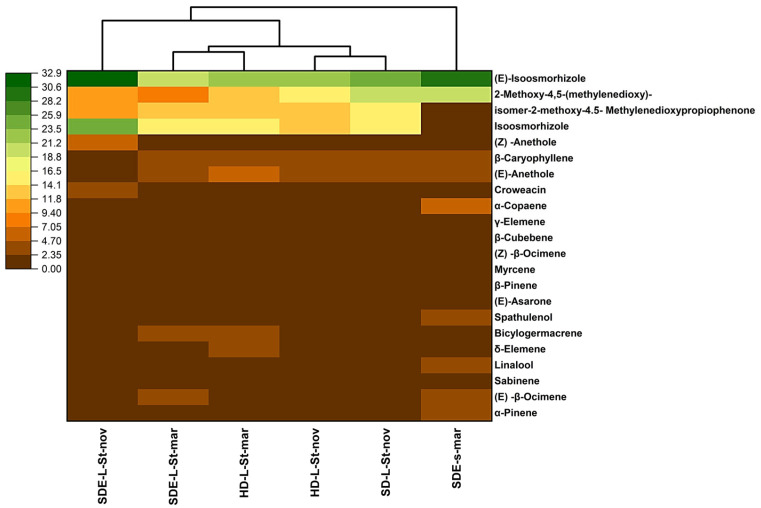
Heatmap clustering consisting of hierarchical cluster analysis (HCA) of the samples under investigation based on their chemical constituents.

Heatmap clustering shows the closeness of the samples on the basis of their chemical composition (Figure 1). In the heatmap, only compounds >1.0% are considered. In the color gradient, yellow represents the lowest constituent percentage, and green represents the highest constituent percentage. HCA showed three major clusters. In the first cluster, only SDE-L-St-nov was present, while the second cluster consisted of four samples, i.e., SDE-L-St-Mar, HD-L-St-Mar, HD-L-St-Nov, and SD-L-St-Nov. The third cluster consisted of only one sample, i.e., SDE-s-Mar.

**Figure 2:**
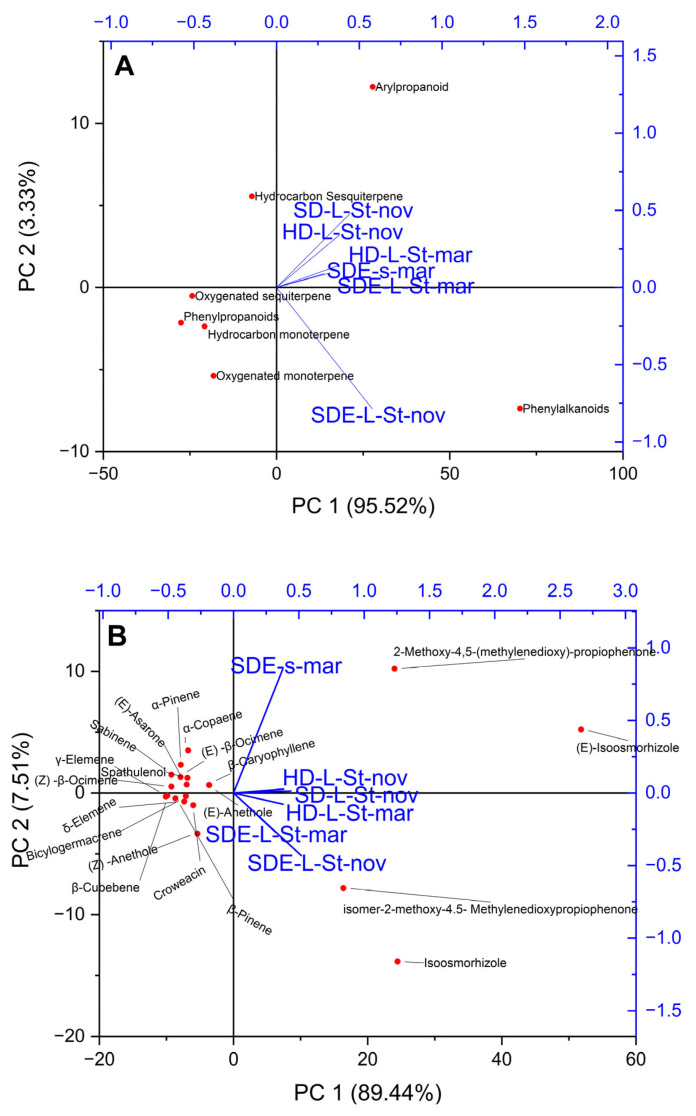
(**A**) Principal component projection for the factors (1B, major constituents) that have the greatest effects on the plants under study. (Dependent variables are shown as blue segments, while mean values are plotted as dots.) (**B**) Principal component projection for the factors (1B, major constituents) that have the greatest effects on the plants under study. (Dependent variables are shown as blue segments, while mean values are plotted as dots.)

A multivariate statistical approach, PCA, was performed to distinguish the studied samples based on the class of compounds and major chemical constituents (>1.0%). The first two principal components (PCs) explained over 97% of the total variance. SDE-L-St-nov was separated by phenylalkaloids (Figure 2A). Among the chemical constituents, the samples were mainly separated by 2-methoxy-4,5-(methylenedioxy)-propiophenone, (E)-isoosmorhizole, 2-methoxy-4.5-methylenedioxypropiophenone isomer, and isoosmorhizole (Figure 2B). The HCA results also supported the PCA results.

### 2.3. Antioxidant Activity

The Trolox antioxidant standard was similarly used to test the samples. A concentration versus inhibition curve was prepared to directly compare the standard with the samples. The inhibition curve of Trolox was prepared at concentrations of 10.0–1 µg·mL^−1^, and the inhibition varied from 84.6% to 12.2%, as observed in Table 3.

The reaction was quite fast, approximately 10 min. The dose–response correlation was highly linear (R^2^ = 0.97), and the obtained linear equation (y = 0.108x) was used to express the antioxidant activity results in mg of Trolox equivalents per mL of oil (mg TE·mL^−1^). The EOs of *P. marginatum* were evaluated at a single concentration; the end point of the reaction was determined after 120 min; the absorbance was measured at 517 nm; and the results are expressed in terms of Trolox equivalents, as shown in Table 4.

The tested oils exhibited DPPH inhibition ranging from 31.2 ± 1.5 to 49.8 ± 3.0%. The EOs of the leaves and stems of *P. marginatum* extracted by SD in November and the EOs of the leaves extracted by HD in March showed the highest antioxidant capacity. Regarding antioxidant activity, the EOs of the leaves and stems obtained by HD in November had the highest value (135.3 mg TE·mL^−1^), followed by the EOs of the leaves and stems extracted by HD in March (126.8 mg TE·mL^−1^). The EOs of the leaves and stems of *P. marginatum* obtained by SD in November exhibited lower potential (84.9 mg TE·mL^−1^) than the other EO fractions studied. However, the percentage of antioxidant activities was significantly higher than that found in the EO of the roots of *P. marginatum* collected in the state of Rondônia, except for the EO extracted from the leaves and stems in March [44].

### 2.4. Preliminary Toxicity

In the control group, no mortality was observed. This demonstrates that it is feasible to use dimethyl sulfoxide (DMSO) as a solvent for bioassays with *A. salina* larvae. The LC_50_ values were calculated by converting the percentage of larval mortality into probit values [51], which were used to draw a linear equation on a semilogarithmic scale. The results of the present work can be observed in Table 5; in all analyzed cases, the concentrations that presented mortality were those superior to 10 µg·mL^−1^. The analyzed samples Pm-SD-L-St-Nov and Pm-HD-L-St-Nov showed mortality above 80% at concentrations of 25 µg·mL^−1^, and the LC_50_ was 17.47 ± 0.33 µg·mL^−1^ and 17.33 ± 0.53 µg·mL^−1^, respectively. Values above the positive control lapachol had an LC_50_ of 21.2 ± 2.2 µg·mL^−1^, that is, presenting a superior bioactivity. According to Meyer et al. [52], an extract can be considered toxic if its LC_50_ value is ≤30 µg·mL^−1^. In the present results, this may be associated with the presence of a class of compounds such as phenylpropanoids and phenylalkanoids [53,54,55] or, more specifically, may be related to the presence of the compounds (*E*)-Anethole, Isoosmorhizole, (*E*)-Isoosmorhizole, iso-mer-2-methoxy-4.5-Methylenedioxypropiophenone, and 2-Methoxy-4,5-(methylenedioxy)-propiophenone, as shown in Table 2.

### 2.5. Analysis of the Interactions of Major Compounds with AChE

#### 2.5.1. Molecular Docking Analysis

**Figure 3:**
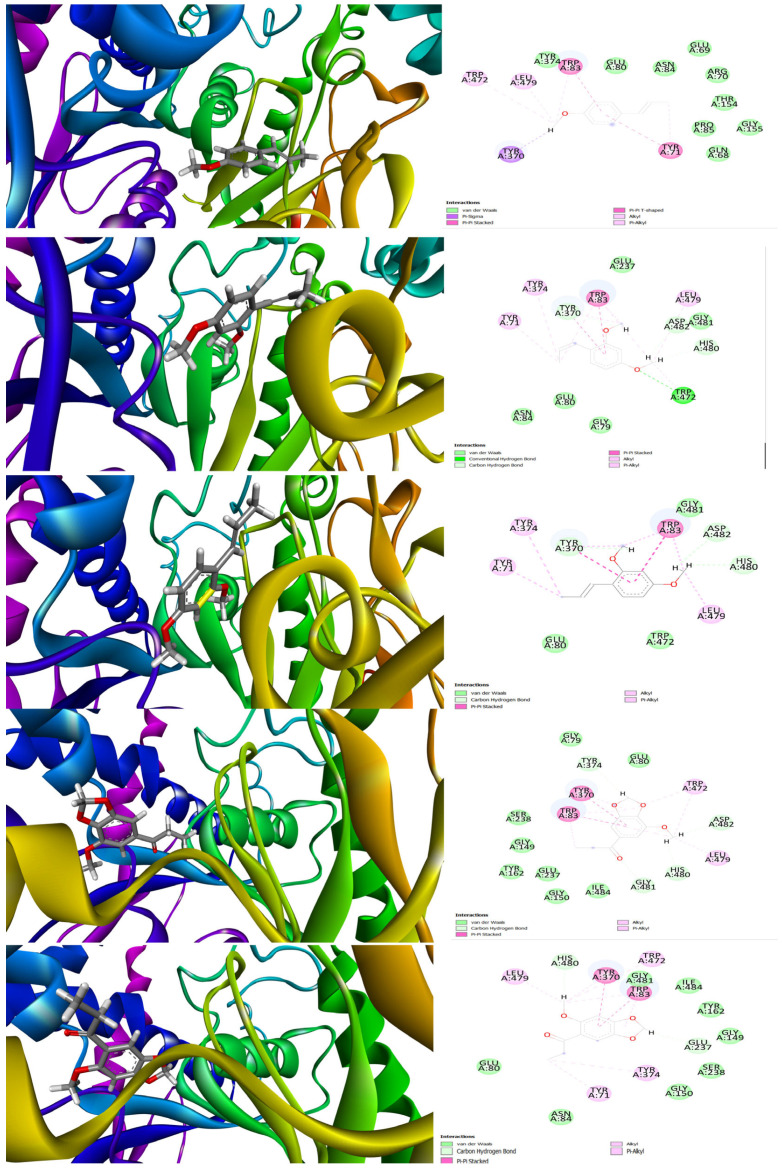
Three-dimensional- and two-dimensional-interaction diagrams for docked ligands, (E)-Anethole, Isoosmorhizole, (E)-Isoosmorhizole, isomer-2-methoxy-4.5-Methylenedioxypropiophenone, and 2-Methoxy-4,5-(methylenedioxy)-propiophenone against *AChE*.

The interaction between the molecules of natural origin and the molecular targets of pharmacological interest was effectively assessed using in silico methods [34]. The present research employed molecular docking to analyze the interaction of the principal compounds found in specific plant species with the binding pocket of AChE, which is a molecular target that is associated with toxicity and was previously investigated in A. salina models [34]. Thus, we tested five major components such as (*E*)-Anethole, Isoosmorhizole, (*E*)-Isoosmorhizole, isomer-2-methoxy-4.5-Methylenedioxypropiophenone, and 2-Methoxy-4,5-(methylenedioxy)-propiophenone against the binding cavity of AChE (Figure 3). Our molecular docking results suggested that the compound (*E*)-Isoosmorhizole exhibited the best docking score, −9.76 kcal/mol, compared to the other essential oil (EO) components. The compound (*E*)-Anethole remained interacting with the active binding site amino acid residues via π–Sigma, π–π stacking, van der Waals force, and alkyl or via π–alkyl interactions (docking score of −8.11 kcal/mol). The key amino acid residues involved during docking were Tyr 71, Trp 83, Tyr 370, Leu 479, Trp 472, Pro 85, etc. Isoosmorhizole had a conventional hydrogen bond interaction with amino acid Trp 472, while other residues, Tyr 370, His 480, and Asp 482, showed van der Waals-type interactions. Amino acid residues such as Leu 479, Trp 83, Tyr 374, Tyr 71, and Tyr 370 were indicated as being mostly alkyl or via π–alkyl interactions for (*E*)-Isoosmorhizole (docking score of −9.10 kcal/mol). For isomer-2-methoxy-4.5-Methylenedioxypropiophenone and 2-Methoxy-4,5-(methylenedioxy)-propiophenone, we noticed key interactions with Tyr 370, Trp 83, Trp 472, Leu 479, His 480, Glu 237, Gly 149, Ser 238, Tyr 162, etc. (docking scores of −7.89 and −8.34 kcal/mol, respectively).

#### 2.5.2. Molecular Dynamics Analysis (MDA)

**Figure 4:**
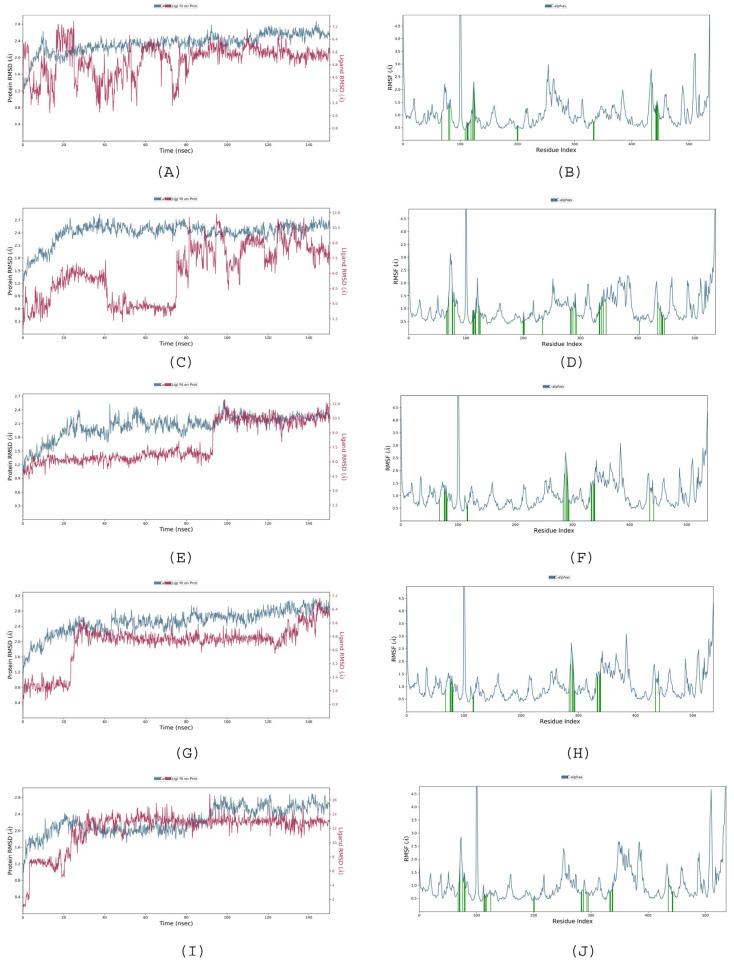
RMSD (**A**,**C**,**E**,**G**,**I**) and RMSF (**B**,**D**,**F**,**H**,**J**) diagrams for ligands, (*E*)-Anethole, Isoosmorhizole, (*E*)-Isoosmorhizole, isomer-2-methoxy-4.5-Methylenedioxypropiophenone, and 2-Methoxy-4,5-(methylenedioxy)-propiophenone, respectively, against *AChE* simulated over the period of 150 ns.

**Figure 5:**
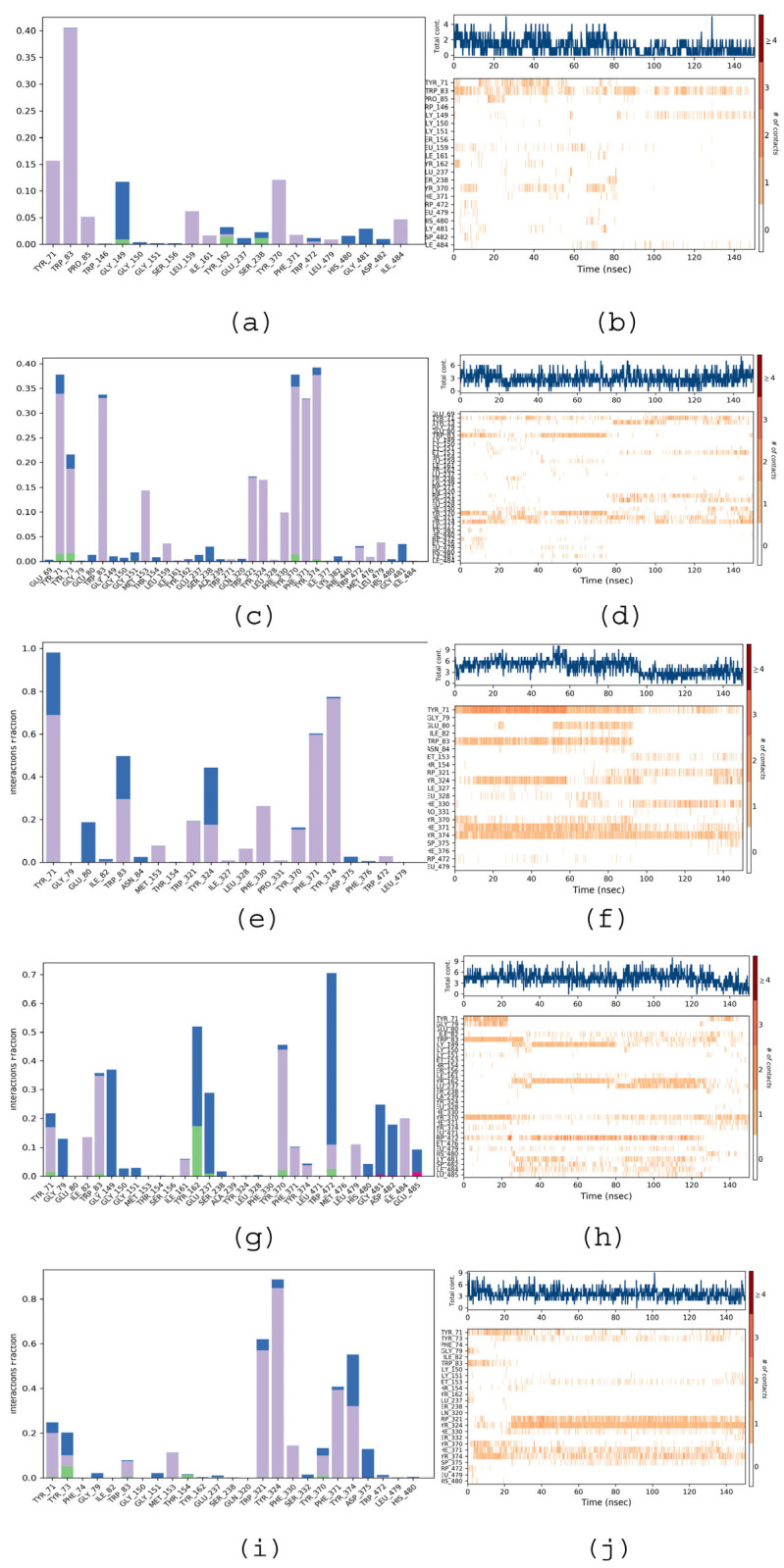
Protein–ligand interaction profile (**a**,**c**,**e**,**g**,**i**) and protein–ligand interaction timeline (**b**,**d**,**f**,**h**,**j**) diagrams for ligands, (*E*)-Anethole, Isoosmorhizole, (*E*)-Isoosmorhizole, isomer-2-methoxy-4.5-Methylenedioxypropiophenone, and 2-Methoxy-4,5-(methylenedioxy)-propiophenone, respectively, against *AChE* simulated over the period of 150 ns.

The stability and convergence of analyses for acetylcholinesterase (AChE target) against various EO components ((*E*)-Anethole, Isoosmorhizole, (*E*)-Isoosmorhizole, isomer-2-methoxy-4.5-Methylenedioxypropiophenone, and 2-Methoxy-4,5-(methylenedioxy)-propiophenone) were analyzed using extended molecular dynamics simulations over the period of 150 ns using the “Desmond, Schrodinger, LLC, NY, 2022” tool [6]. The results of the 150 ns simulation indicated a consistent conformation, as evidenced by the root-mean-square deviation (RMSD) analysis. From Figure 4A–I, it is very clear that complexes with EO components had a stable RMSD value. The RMSD of the backbone of the (*E*)-Isoosmorhizole-AChE C-RMSD complex remained under 3.2 Å, while the ligand RMSD of (E)-Isoosmorhizole was 6.4 Å, indicating good convergence and stable conformations throughout the simulation (Figure 4E). This conclusion is further corroborated by the stable graphs of the root-mean-square deviation (RMSD). The ligand’s high binding affinity implies a stable complex with AChE, as evidenced by Figure 2B–J. While there were some minor fluctuations in the the root-mean-square (RMSF) fluctuations plot for AChE, most of the residues remained relatively constant throughout the simulation. This suggests that the protein structure is stiff in the ligand-bound conformations and that the residues may be more flexible, as indicated by the RMSF plots. The RMSF values of the EO components, including (*E*)-Anethole, Isoosmorhizole, (*E*)-Isoosmorhizole, isomer-2-methoxy-4.5-Methylenedioxypropiophenone, and 2-Methoxy-4,5-(methylenedioxy)-propiophenone, showed noticeable but minimal fluctuations. These fluctuations indicate that these ligands exhibit significant internal atomic fluctuations during their interaction with AChE, which can be attributed to their flexibility properties. The flexibility of these small molecule ligands allows them to adopt various conformations and interaction patterns within the receptor protein cavity, resulting in the observed fluctuations in the RMSF values. Overall, the results of the simulation suggest that the complex formed between (E)-Isoosmorhizole and AChE is stable and that the amino acid conformations are also stable. The considerable decrease in gyration (Rg) signifies that the protein assumes a tightly aligned configuration upon binding to the ligand. Additionally, the existence of hydrogen bonds between the protein and ligand supports the notion that the complex is both stable and has a strong interaction. During the 150 ns simulation, the compound (E)-Isoosmorhizole and AChE were observed to form significant hydrogen bonds (Figure 5e), and oppositely charged residues also exhibited salt bridges, which significantly contributed to the protein’s stability. Collectively, the analysis of Rg suggests that the protein structure becomes more condensed and less pliant following ligand binding.

#### 2.5.3. Protein—Ligand Interactions

Figure 3a–j illustrate the interactions that occurred between the amino acid residues of AChE and the ligands during the simulation time (0.00–150.00 ns). The “protein–ligand contact” plots indicate the time fractions of protein–ligand interactions that were maintained throughout the simulation. The “Timeline of interactions and contacts” diagram shows the timelines of interactions and contacts, including H-bonds and hydrophobic, ionic, and water bridges. The top panel displays the total number of specific contacts between the protein and the ligand during the simulation time. The bottom panel provides a detailed list of the residues that interacted with the ligand in each frame of the MD simulation course. Residues with multiple contacts with the ligand are represented by darker shades of orange, as indicated on the right side of the diagram.

From Figure 5a, we can notice that compound (*E*)-Anethole in the AChE complex is stabilized via H-bond, hydrophobic, and water-bridge-like interactions. Tyr 71, Trp 83, Pro 85, Leu 159, Ile 161, Tyr 370, and Phe 371 residues mainly depicted hydrophobic interactions during the entire 150 ns simulation period. According to Figure 3c (Isoosmorhizole–AChE complex), the stability of the ligand Isoosmorhizole with the AChE is largely due to hydrophobic interactions with the amino acid residues Tyr 71, Tyr 73, Trp 83, Met 153, Leu 159, Gln 320, Trp 321, Phe 330, Tyr 370, Tyr 374, Leu 479, etc. According to Figure 5e ((E)-Isoosmorhizole-AChE complex), the majority of contacts were hydrophobic interactions with residues Tyr 71, Trp 83, Met 153, Trp 321, Tyr 324, Ile 327, Phe 330, Phe 371, Tyr 374, and Trp 472 as well as water-bridging interactions with Tyr 71, Glu 80, Trp 83, Tyr 324, and Asp 375. For other complexes (Figure 5g,i) (the isomer-2-methoxy-4.5-Methylenedioxypropiophenone- AChE complex and the 2-Methoxy-4,5-(methylenedioxy)-propiophenone-AChE complex), we observed the majority of the water-bridging interactions with Tyr 71, Gly 79, Gly 151, Glu 237, Tyr 370, and Trp 472.

## 3. Materials and Methods

### 3.1. Collection of Botanical Material

The specimens (*P. marginatum* sensu lato) were collected on the campus of the Federal Rural University of Amazonia in the city of Belém, Pará, in the morning on days between November 2018 and March 2019. Botanical identification was performed by comparison with materials identified by Elsie Franklin Guimarães, a specialist in Piperaceae, and samples were incorporated into the “João Murça Pires” Herbarium of the Emílio Goeldi Museum of Pará (*P. marginatum* sensu lato MG184836) in Belém, Pará.

### 3.2. Determination of Residual Moisture

Before moisture analysis, the sample was dried in an air circulation oven at approximately 35 °C, for a period of 5 days. The moisture present in the samples was determined with the aid of a Gehaka infrared moisture analyzer (IV2500).

### 3.3. Essential Oil Extraction

#### 3.3.1. Hydrodistillation

For the EO extraction process, 40 g of fresh botanical material were dried in an air circulation oven and then subjected to HD. Equal proportions of water and plant material were used, according to the methodology described by Ferreira et al. [56]. Essential oils from leaves and stem were not separately extracted, and the process was carried out for 3 h, at a temperature of approximately 100 °C.

#### 3.3.2. Distillation and Simultaneous Extraction

Distillation–simultaneous extraction was performed in a Chrompack Nickerson and Likens extractor coupled to a refrigeration system (5–10 °C) and connected to two round-bottomed flasks. Then, 10 g of botanical material and 125 mL of distilled water were added to a 250 mL flask with a heating mantle, from which the vapors passed to the condenser. Two milliliters of n-pentane were added to a 5 mL flask, which was kept in a water bath at 53–56 °C for evaporation and extraction (condensation) of the volatile concentrate. The extraction time was 2 h [57].

#### 3.3.3. Steam Distillation

The EO isolation process using steam distillation (SD) was carried out using a modified Clevenger glass system apparatus coupled to a refrigeration system to maintain the condensation water between 10 and 15 °C for 3 h, as described by [38].

### 3.4. Identification of Chemical Constituents

The chemical compositions of the *P. marginatum* EO samples were analyzed using a single quadrupole gas chromatography/mass spectrometry (GC/MS) system (Thermo DSQ-II, Waltham, MA, USA) equipped with a DB-5MS silica capillary column (30 m × 0.25 mm, 0.25 mm; Agilent Technologies, Stevens Creek Blvd., Santa Clara, CA, USA). Aqueous 2:1000 n-hexane was injected in one step (0.1 mL); the temperature of the ion source and other parts was set at 200 °C. The operational conditions of injection and identification were previously described by our research group [57]. The components were identified by comparison of (i) the experimental mass spectra with those compiled in libraries (reference) and (ii) the experimental retention indices with those found in the literature [49,50]. The volatile constituents were quantified by peak-area normalization using a FOCUS GC/flame ionization detector (FID), which was operated under the same conditions as the GC–MS instrument.

### 3.5. Antioxidant Potential

The essential oil samples (10 μL) were mixed with 900 μL of 100 mM Tris-HCl buffer (pH = 7.4), 40 μL of ethanol, and 50 μL of a 0.5% Tween 20 solution (m/m), and then 1.5 mL of 0.5 mM DPPH in ethanol (250 μM in the reaction mixture) was added. Tween 20 was used as an emulsifier for oil–water mixing [58,59]. The mixture was vigorously stirred and kept in a dark environment at room temperature for 30 min. The absorbance reading was performed in the UV–visible at 517 nm in an 800XI spectrophotometer (Femto; São Paulo/SP, Brazil). The control reaction was performed by replacing the sample with 50 μL of Trolox 1 mM in ethanol (the final concentration in the reaction was 25 μM). Calculation of inhibition percentage—IDPPH (%). The percentage of inhibition of DPPH radicals (IDPPH) was performed according to what is described in the literature [59]. The percentages of inhibition of the oils were compared with the inhibition induced by the 1 mM Trolox solution. The total antioxidant capacity expressed in mg ET/mL of oil was calculated according to the equation proposed by [60,61]. Essential oils were tested without dilution.

### 3.6. Determination of Preliminary Toxicity in Artemia salina Leach

An artificial brine was prepared with 46 g of NaCl, 22 g of MgCl2·6H2O, 8 g of Na_2_SO_4_, 2.6 g of CaCl_2_·_2_H_2_O or CaCl_2_·_6_H_2_O, and 1.4 g of KCl dissolved in 2000 mL of distilled water. The brine pH was adjusted to 9.0 using Na_2_CO_3_ to avoid the risk of larval death due to a pH decrease during the incubation period [62].

*A. salina* cysts (25 mg) were incubated in artificial brine at 25 °C in a glass container with a capacity of 10.6 dm3 and an oxygenation system consisting of an aeration pump. The container consisted of two parts, one containing the eggs, which was protected from light, and one that was illuminated by artificial light generated by a 40 W lamp.

This division was performed because the larvae have positive phototropism, i.e., affinity for light, and, consequently, after hatching, the larvae migrated through the partition to the illuminated portion of the glass container and were separated [62,63].

The EO solution was prepared at a concentration of 1250 μg·mL^−1^ using brine water (without larvae) as the solvent and 5% dimethyl sulfoxide (DMSO) as the solubilizer. Aliquots of the stock solution were diluted to concentrations of 1, 5, 10, 25, 50, 100, 250, 500, and 1000 μg·mL^−1^.

Twenty-four hours after hatching, approximately 10 larvae were added to the sample test tubes using an automatic micropipette. The tubes were filled to a total volume of 5 mL with brine water. The control group was prepared using 5 mL of 5% DMSO brine and 10 *A. salina* larvae. The experiments were performed in triplicate (n = 3).

After 24 h of contact between the *A. salina* larvae and the sample solutions, the percent mortality was calculated. The LC_50_ value was calculated using semilogarithmic interpolation by converting mortality percentages into probits [62]. For the control, a naphthoquinone extracted from the bark of several species of plants of the genus *Tabebuia* (Bignoniaceae), lapachol, which has wide biological activity against different organisms, was used as a positive standard [64].

### 3.7. In Silico Analysis (Molecular Docking and Molecular Dynamics)

To investigate how 5 compounds, (*E*)-Anethole, Isoosmorhizole, (*E*)-Isoosmorhizole, isomer-2-methoxy-4.5-Methylenedioxypropiophenone, and 2-Methoxy-4,5-(methylenedioxy)-propiophenone), interact with the binding cavity of the acetylcholinesterase (AChE) protein, molecular docking was performed. The compounds were drawn using GaussView 6 and optimized via B3LYP/6-31G* using Gaussian quantum chemistry software 16 [34,65]. Glide software from Schrodinger, LLC, 2023 [66,67] was used for the docking process, with the crystal structure of the AChE obtained from the Protein Data Bank (https://www.rcsb.org/, accessed on 25 May 2023). The binding free energy was measured using Prime, and visualizations were completed using Chimera v15 and BIOVIA Discovery Studio Visualizer v21.1.

Next, molecular dynamics simulations were carried out on the docked complexes using the Desmond 2022 software from Schrodinger, LLC. The OPLS-2005 force field [68,69] and TIP3P water molecules were used in an explicit solvent model [70], and Na^+^ ions were added to balance the charge. NaCl solutions were also included to mimic physiological conditions. The system was equilibrated using an NVT ensemble for 10 ns and an NPT ensemble for 12 ns. The Nose–Hoover chain coupling approach was used to set up the NPT ensemble and the variable temperature. During the simulations, a time step of 2fs was used, and the Martyna–Tuckerman–Klein chain coupling technique was used to manage pressure [71]. The final production run lasted for 100 ns, and the stability of the MD simulations was monitored using root-mean-square deviation (RMSD), radius of gyration (Rg), root-mean-square fluctuation (RMSF), H-bonds, salt bridges, and SASA calculations [72].

### 3.8. Statistical Analysis

Chemometric analysis was performed on the basis of two multivariate analyses, i.e., heatmap clustering consisting of hierarchical cluster analysis (HCA) and principal component analysis (PCA). Multivariate analyses were performed using OriginPro 2023 version 10.0.0.154 (Learning Edition).

## 4. Conclusions

In the present work, it was observed that the class of phenylpropanoid compounds, and the major substances isoosmorhizole, (E)-Isoosmorhizole, isomer-2-Methoxy-4.5-methylenedioxypropiophenone, and 2-Methoxy-4,5-(methylenedioxy)-propiophenone, may be responsible for the potential toxicity of antioxidants. In general, natural antioxidants may be promising inhibitors of oxidative stress; however, it is important that the toxicity of these molecules be low even in small concentrations. Furthermore, the results of in silico studies demonstrated that the main constituents present in the essential oil fractions interact with the molecular target (catalytic site) of AChE. This is the first report on the molecular interaction of the compounds present in the essential oils of *P. marginatum* in AChE; in addition, this *P. marginatum* chemotype may be an important source of bioactive compounds, so further studies need to be carried out to explore the biological potential of this species. We also recommend studies in human cells to measure the toxicity of this essential oil.

## Figures and Tables

**Table 1:** Yields of essential oils extracted by different methods. **HD**: hydrodistillation; **SD**: steam distillation; **L**: leaves; **St**: stems; **Nov**: November; **Mar**: March.

*Piper marginatum*			
	HD	SD	
	L-St-Nov	L-St-Mar	L-St-Nov
Mass EO (g)	0.59	0.66	0.53
* Yields (%)	1.66	1.83	1.49
Moisture (%)	11.1	9.0	11.1

* Yields in % (mass of essential oils/mass of moisture-free sample).

**Table 3:** Trolox standard and concentrations.

Sample	Concentration (µg·mL^−1^)	Inhibition (%)
**Trolox**	10	84.6 ± 1.8
	5	53.4 ± 2.0
	2.5	29.8 ± 1.9
	1	12.2 ± 3.6

**Table 4:** DPPH sequestration activity of the essential oils of *Piper marginatum* (**Pm**) (%).

Sample (EO)	Inhibition	mg TE·mL^−1^
**Pm-SD-L-St-Nov**	31.2 ± 1.5	84.9 ± 4.0
**Pm-HD-L-St-Nov**	49.8 ± 3.0	135.3 ± 8.2
**Pm-HD-L-St-Mar**	46.7 ± 4.5	126.8 ± 12.3

**Pm** = *Piper marginatum;* **HD**: hydrodistillation; **SD**: steam distillation; **L**: leaves; **St**: stems; **Nov**: November; **Mar**: March.

**Table 5:** Preliminary toxicity of essential oil samples of *Piper marginatum*.

Sample	Concentration (µg·mL^−1^)	Mortality (%)	R^2^	LC_50_ (µg·mL^−1^)
**Lapachol**	50	100		(µg·mL^−1^)
	25	66.7		
	10	3.3	0.93	21.2 ± 2.2
	5	0		
**Pm-SD-L-St-Nov**	50	100		
	25	86.6	1	17.47 ± 0.33
	10	10		
	5	0		
**Pm-HD-L-St-Nov**	50	100		
	25	83.3	1	17.33 ± 0.53
	10	16.6		
	5	0		

**Pm** = *Piper marginatum;* **HD**: hydrodistillation; **SD**: steam distillation; **L**: leaves; **St**: stems; **Nov**: November.

## Data Availability

Not applicable.

## Supplementary Materials

The following supporting information can be downloaded at https://www.mdpi.com/article/10.3390/molecules28155814/s1. Figures S1–S6 of ion chromatograms of samples of oils and aromas.

## Sample Availability

Samples of the compounds in Museu Paraense Emílio Goeldi are available from the authors.
