# Supplementary material for: Chemical Composition, Preliminary Toxicity, and Antioxidant Potential of Piper marginatum Sensu Lato Essential Oils and Molecular Modeling Study
Source: Molecules. 2023 Aug 1;28(15):5814. doi: 10.3390/molecules28155814 (PMC10421147; doi:10.3390/molecules28155814)
Supplement: Supplementary file 1 [file molecules-28-05814-s001.zip › molecules-2519512-supplementary.pdf]

Supplementary material S1

Chemical Composition, Preliminary Toxicity, Antioxidant Potential of *Piper marginatum* sensu lato Essential Oils, and Molecular Modeling Study

Bruna de Souza Feitosa [1], Oberdan Oliveira Ferreira [2,4], Suraj N. Mali[3], Amit Anand[3], Jorddy Nevez Cruz[1], Celeste de Jesus Pereira Franco[1], Sonu Kumar Mahawer[4], Ravendra Kumar[4], Marcia Moraes Cascaes[5], Mozaniel Santana de Oliveira[6,7*], and Eloisa Helena de Aguiar Andrade [1,2,5,6,7].

[1]Universidade Federal do Pará, School of Chemistry, Rua Augusto Corrêa S/N, Guamá, 66075-900 Belém, Pará, Brazil

[2]Graduate Program in Biodiversity and Biotechnology–Rede Bionorte, Universidade Federal do Pará, Rua Augusto Corrêa S/N, Guamá, 66075-900 Belém, Pará, Brazil.

[3]Department of Pharmaceutical Sciences and Technology, Birla Institute of Technology, Ranchi 835215, India.

[4]Department of Chemistry, College of Basic Sciences and Humanities, Govind Ballabh Pant University of Agriculture and Technology, Pantnagar 263145, U.S. Nagar, Uttarakhand, India

[5]Graduate Program in Chemistry, Universidade Federal do Pará, Rua Augusto Corrêa S/N, Guamá, 66075-900 Belém, Pará, Brazil.

[6]Programa de Pós-Graduação em Ciências Biológicas - Botânica Tropical, Universidade Federal Rural da Amazônia, and Museu Paraense Emílio Goeldi, Av. Perimetral, 1901, Terra Firme, 66077-830, Belém, PA, Brazil, Brazil.

[7]Adolpho Ducke Laboratory-Coordination of Botany, Museu Paraense Emílio Goeldi, Av. Perimetral, 1901, Terra Firme, 66077-830, Belém, PA, Brazil, Brazil.

*Corresponding author: mozaniel.oliveira@yahoo.com.br or mozanieloliveira@museu-goeldi.br; Phone: + 55 91 988647823.

Abstract: The essential oils (OEs) of the leaves, stems and spikes of P. marginatum were obtained by hydrodistillation, steam distillation and simultaneous extraction. The chemical constituents were identified and quantified by GC/MS and GC-FID. The preliminary biological activity was determined by assessing the toxicity of the samples to *Artemia salina* Leach larvae and calculating the mortality rate and lethal concentration (LC_{50}). The antioxidant activity of the EOs was determined by the DPPH radical scavenging method. Molecular modeling was performed using molecular docking and molecular dynamics, acetylcholinesterase being the molecular target. The OES yields ranged from 1.49 to 1.83%. The EOs and aromatic constituents of *P. marginatum* are characterized by high contents of (*E*)-isoosmorhizole (19.4-32.9%), 2-methoxy-4,5-methylenedioxypropiophenone (9.0-19.9%), isoosmorhizole (1.6-24.5%) and 2-methoxy-4,5-methylenedioxypropiophenone isomer (1.6-14.3%). The antioxidant potential was significant in the OE of the leaves and stems of P. marginatum extracted by SD in November (84.9 ± 4.0 mg TE.mL-1), and the OEs of the leaves extracted by HD in March(126.8 ± 12.3 mg TE.mL-1). Regarding preliminary toxicity, the OEs of Pm-SD-L-St-Nov and Pm-HD-L-St-Nov had mortality higher than 80% in the concentrations of 25 μg.mL-1. The in silico study in essential oils elucidated the potential mechanism of interaction of the main compounds, which may serve as a basis for advances in this line of research.

Keywords: Natural Products; Piperaceae; Amazon; *Artemia Salina;* Volatile Oil; In silico Study.

Ion-chromatograms of essential oils and volatile concentrate of *Piper marginatum*. November collection. **L**: leaves; **St**: stem; **s**: spike; **Nov**: November; **Mar**: March

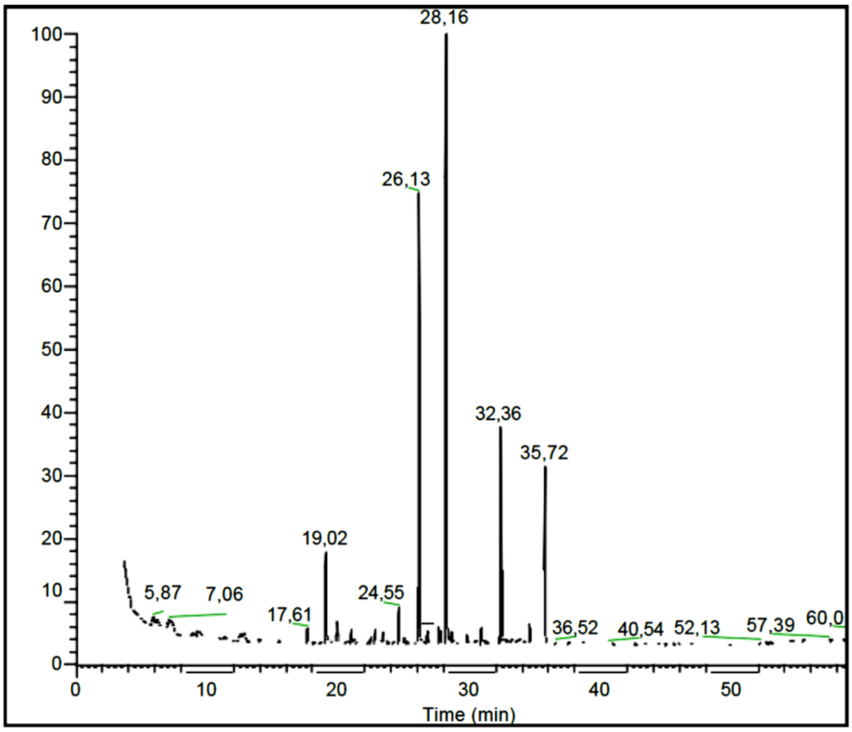

Figure S1. Ion-chromatogram **L-St-Nov** (**SDE**: simultaneous distillation–extraction).

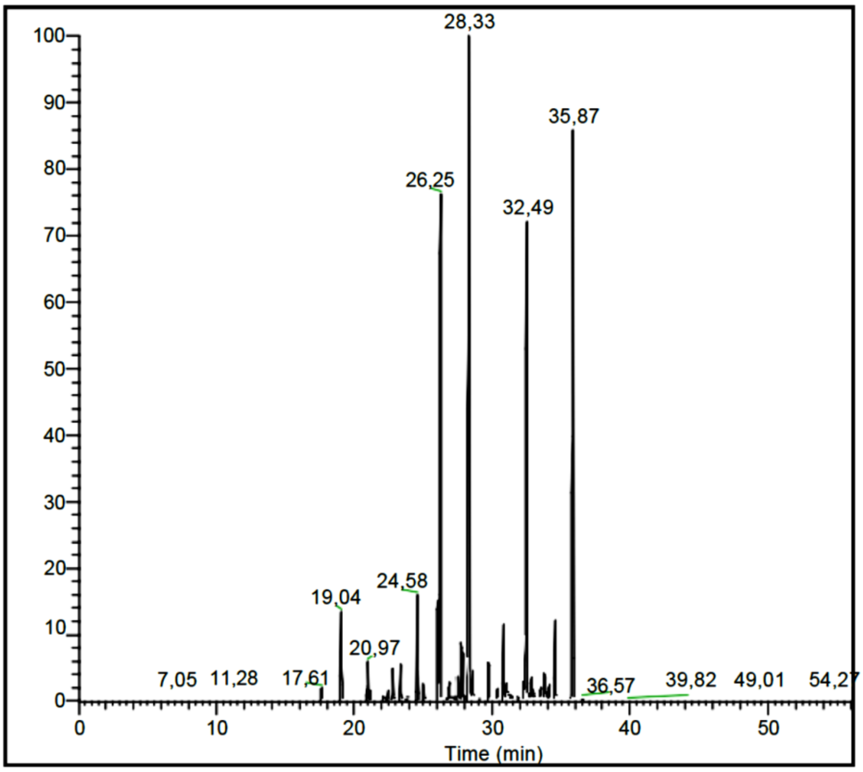

Figure S2. Ion-chromatogram **L-St-Nov** (**SD**: steam distillation).

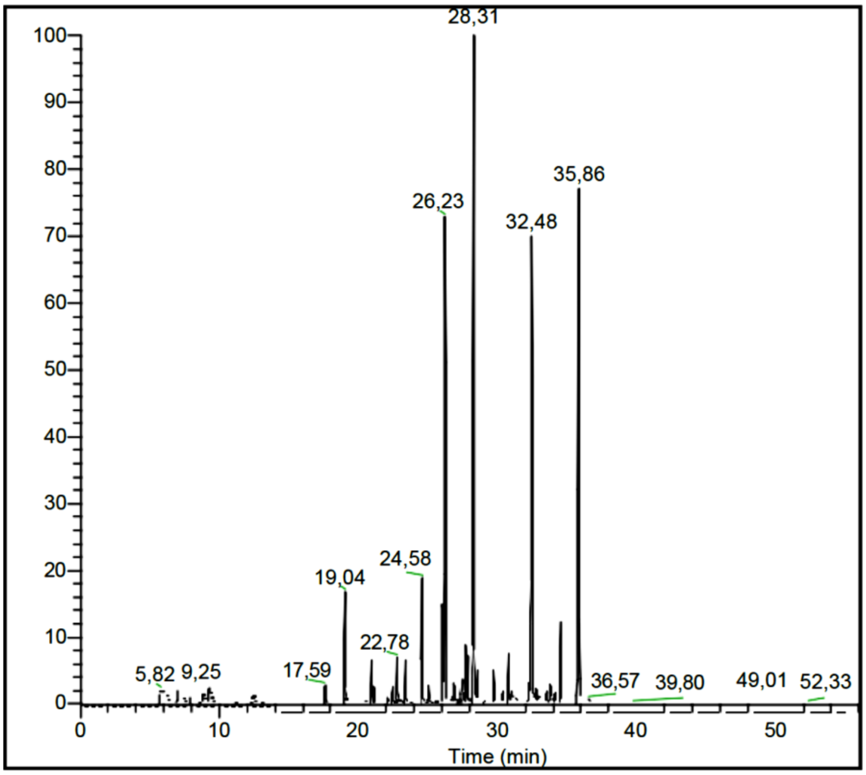

Figure S3. Ion-chromatogram **L-St-Nov (HD**: hydrodistillation).

Ion-chromatograms of essential oils and volatile concentrate of *Piper marginatum,* March collection. **L**: leaves; **St**: stem; **s**: spike; **Nov**: November; **Mar**: March.

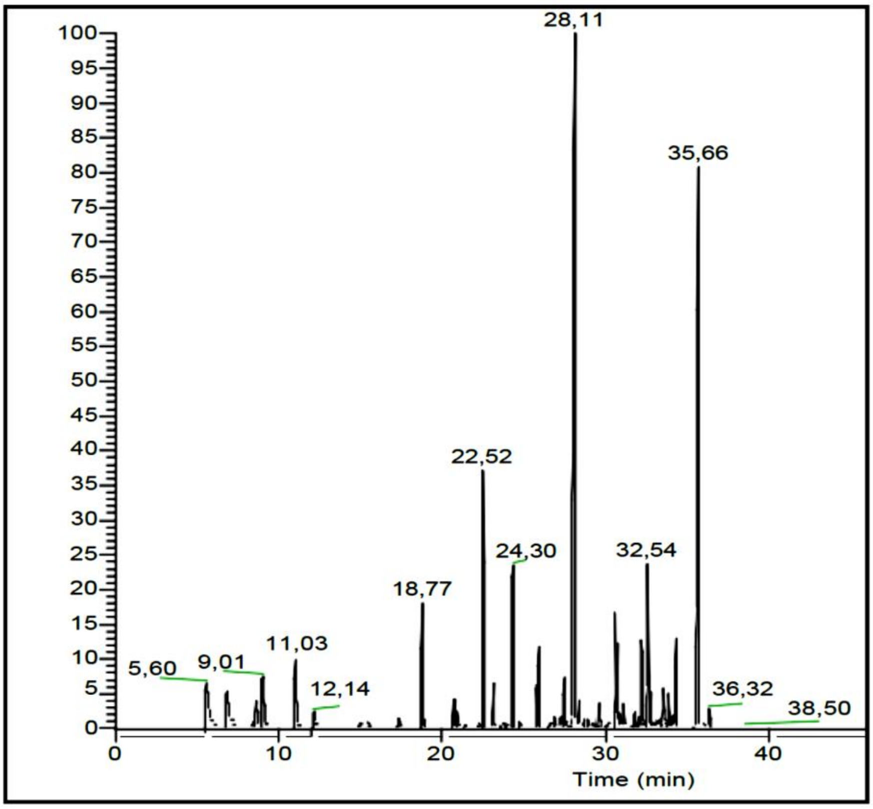

Figure S4. Ion-chromatogram **s-Mar (SDE**: simultaneous distillation–extraction).

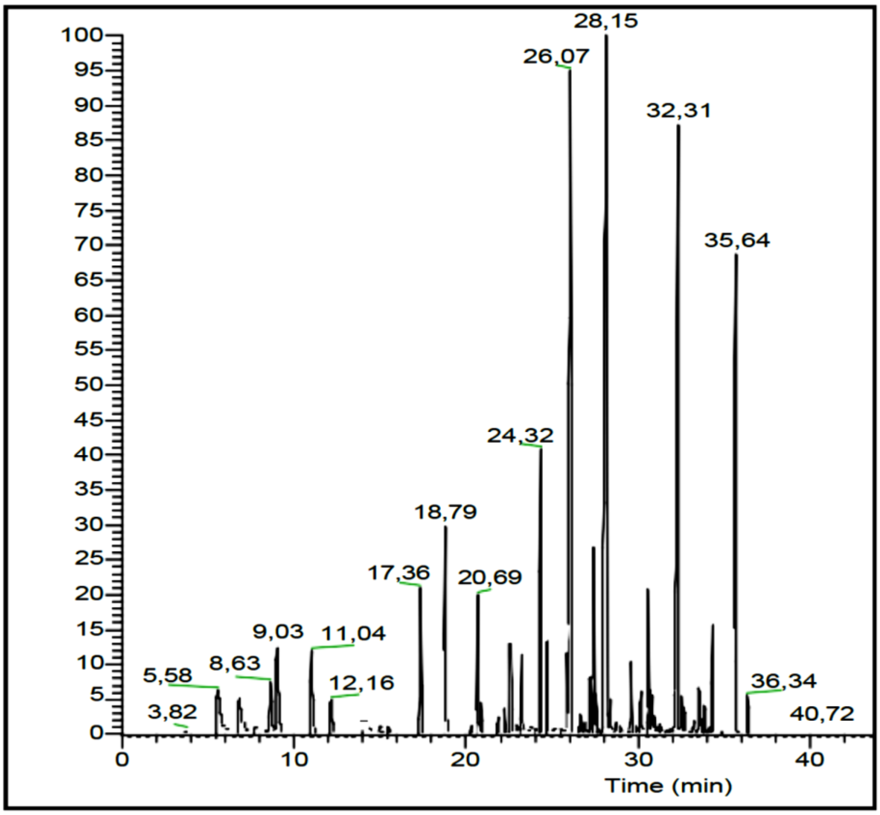

Figure S5. Ion-chromatogram **L-St-Mar** (**SDE**: simultaneous distillation–extraction).

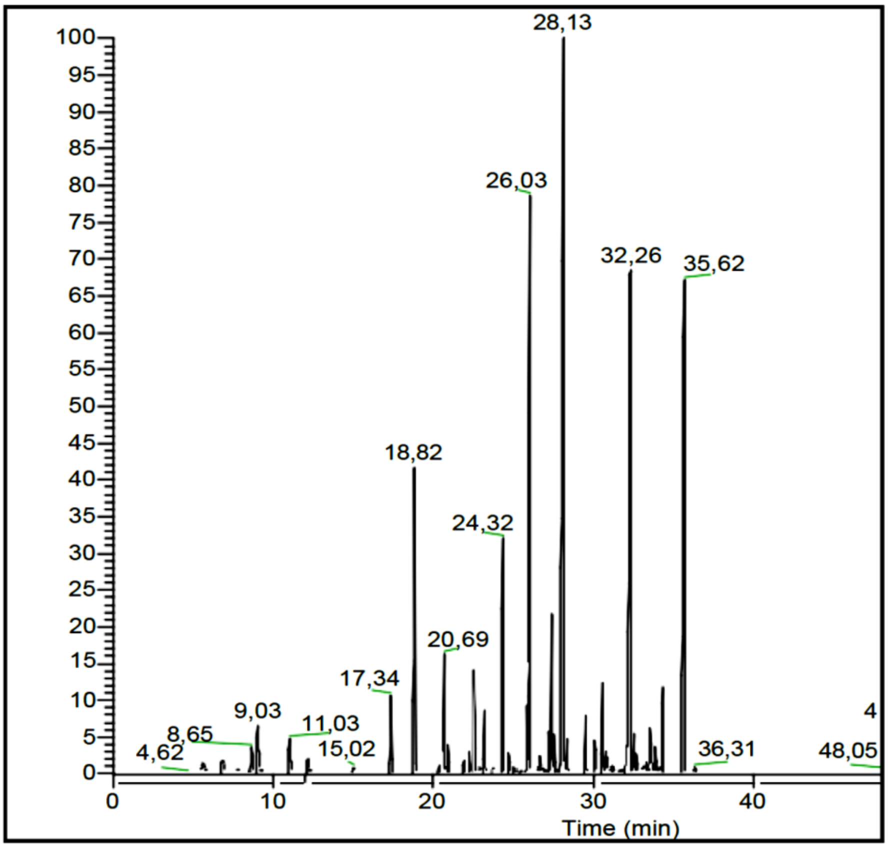

Figure S6. Ion-chromatogram **L-St-Mar (HD**: hydrodistillation).
